# Urogenital schistosomiasis: prevalence, knowledge and practices among women of reproductive age in Northern Tanzania

**DOI:** 10.1016/j.ijregi.2022.09.013

**Published:** 2022-11-28

**Authors:** Neema Ngassa, Abdallah Zacharia, Eliza T. Lupenza, Vivian Mushi, Billy Ngasala

**Affiliations:** Department of Parasitology and Medical Entomology, Muhimbili University of Health and Allied Science, Dar es Salaam, Tanzania

**Keywords:** Urogenital schistosomiasis, Women, Reproductive age, Kileo, Tanzania

## Abstract

**Objective:**

To determine the prevalence, knowledge and practices regarding urogenital schistosomiasis among women of reproductive age in Kileo Ward, Mwanga District.

**Methods:**

A cross-sectional study was conducted in the dispensary in Kileo Village, Mwanga District. A pre-tested structured questionnaire was administered to participants to collect information on sociodemographic characteristics and schistosomiasis-related knowledge and practices. Urine samples were examined for schistosoma eggs using the urine sedimentation technique. Data collected were cleaned, coded and entered into SPSS Version 20 for analysis. Chi-squared test and Fisher's exact test were used for analysis.

**Results:**

Overall prevalence of urogenital schistosomiasis was 2.3%. The prevalence was higher among participants aged ≥18 years (3.2%), those who did domestic chores in river water (13.3%), and those who swam in river water (7.1%). Only a few participants had a low level of knowledge (4.8%) and poor practices (1.9%) regarding urogenital schistosomiasis.

**Conclusion:**

The transmission of urogenital schistosomiasis was low among women of reproductive age in the study area. Despite a high level of knowledge and good practices for urogenital schistosomiasis, there are knowledge gaps regarding its causes and risk factors.

## Highlights

• There has been a significant decrease in urogenital schistosomiasis in the area.
• The findings suggest that the disease is still being transmitted in the area.
• There is a knowledge gap on the causes and risk factors of schistosomiasis.

## Introduction

Urogenital schistosomiasis caused by Schistosoma haematobium is endemic in Africa, the Middle East and Corsica (France). *S. haematobium* is the most prevalent species in Africa, and causes approximately 112 million cases per year. It is estimated that 71 million of these infected individuals experience haematuria (blood in the urine), half of whom have dysuria, and approximately 18 million suffer from urinary bladder pathology. The current best estimate is that kidney failure due to *S. haematobium* infection is responsible for approximately 160,000 deaths each year in Africa [20]. Women living in poor and rural communities, particularly fishing and agricultural areas, are among the vulnerable populations affected by urogenital schistosomiasis. They become infected when doing their daily domestic chores in infested water, such as washing clothes and fetching water [1]. In women, urogenital schistosomiasis may present as genital lesions, vaginal bleeding, pain during sexual intercourse, and nodules in the vulva [21]. Also, women with urogenital schistosomiasis are at increased risk of human immunodeficiency virus (HIV) infection due to immunological activation and lesions (inflammation) developed in the urogenital system by the schistosoma eggs. These lesions thin the epithelium, enabling the agents of sexually transmitted diseases, such as HIV, to pass and have direct access to the systemic circulation [18].

The schistosomiasis control strategy in many African countries (including Tanzania) relies on preventive chemotherapy with praziquantel through mass drug administration to school-aged children [10,21]. However, preventive chemotherapy alone has failed to protect treated individuals from re-infection [22]. Factors such as poor sanitation status, inadequate supply of safe water, lack of information, and risky water contact practices have been associated with the persistent transmission of schistosomiasis in Africa [2]. Therefore, countries should adopt strategies that integrate preventive chemotherapy with other primary healthcare interventions, such as effective health education programmes. Health education is one of the significant components of any disease control programme [14]. To yield sustainable positive changes in any health education promotion, selecting the most appropriate target population is of paramount significance. In Africa, health education interventions on schistosomiasis prevention and control mainly target school-aged children [14,16] as they are considered to be at highest risk of schistosomiasis [9] and are easy to reach [14]. However, unlike other vulnerable groups, women perform most of the water contact activities within the family, putting them at high risk of schistosomiasis. In addition, women are usually the caretakers of other vulnerable groups, such as preschool and school-aged children, and they have an influence on schistosomiasis transmission in these groups [9]. Hence, for effective and successful schistosomiasis control and prevention interventions, women should be considered as one of the important target groups. There is a need to improve understanding of the burden, knowledge and practices of women regarding schistosomiasis in order to design effective control and prevention strategies.

Kileo and Kivulini, two villages in Mwanga District, are endemic for both intestinal and urogenital schistosomiasis, caused by S. mansoni and S. haematobium, respectively. High levels of disease have been reported among school-aged children and women of reproductive age [11,12]. A study of women of reproductive age reported a high prevalence of urogenital schistosomiasis in the area, and a high prevalence of female genital schistosomiasis and its associated genital lesions [11]. In this area, women were observed to be at higher risk of infections compared with other vulnerable groups due to their high-risk water contact practices [12]. Several interventions, such as mass treatment of primary school-aged children and villagers, health education of village leaders and school-going children, improvement of sanitation, and access to safe water, have been undertaken [12]. However, no interventions have been designed specifically for women in the area. Also, no studies have re-evaluated the burden of urogenital schistosomiasis among women of reproductive age in the area since the last study which was conducted more than two decades ago. As such, the present study was conducted to determine prevalence, knowledge and practices regarding urogenital schistosomiasis among women of reproductive age in Kileo Ward, Mwanga District.

## Methods

### Description of the study site

**Figure 1:**
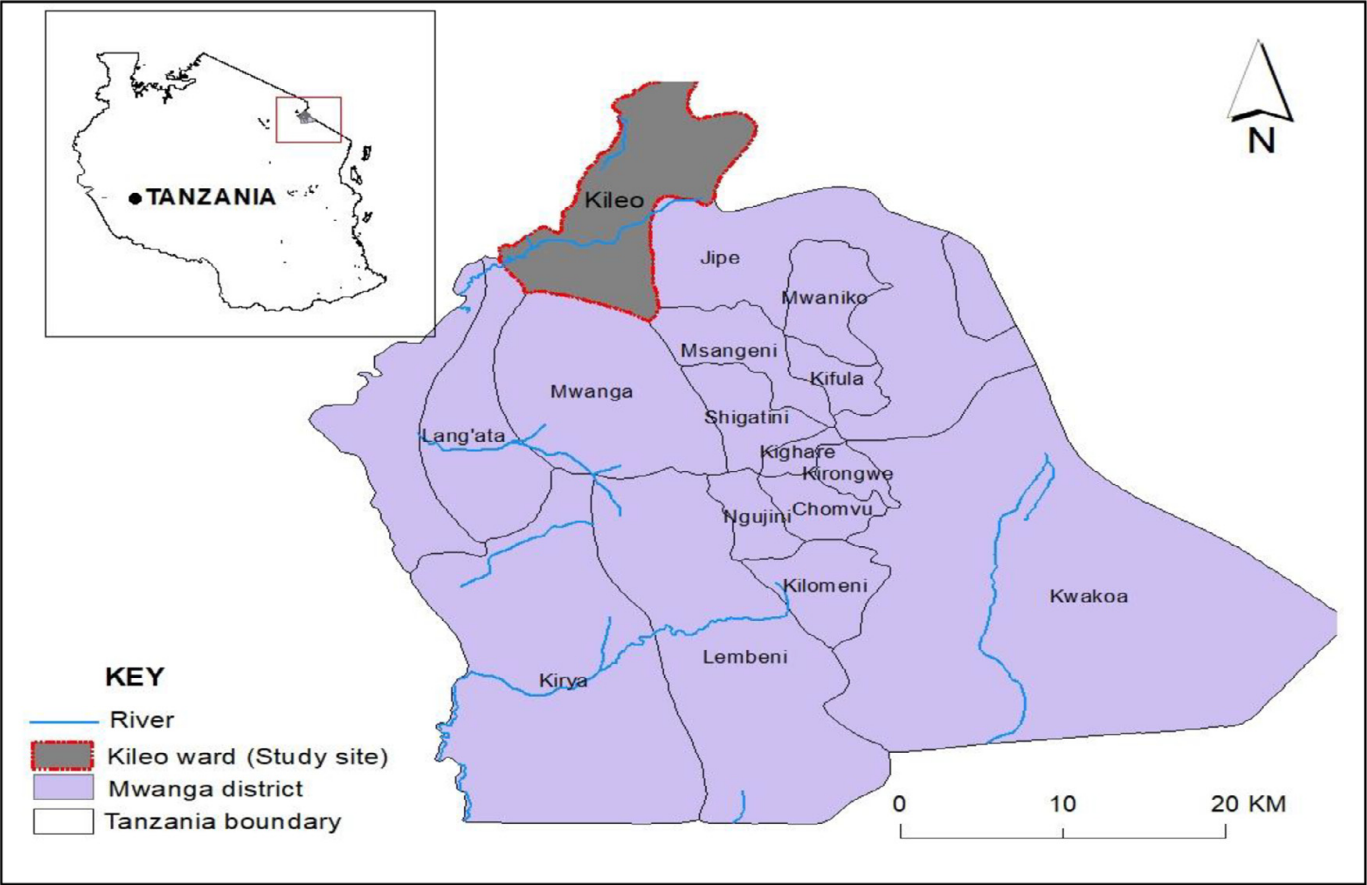
Map of Mwanga District showing Kileo Ward where Kileo and Kivulini villages are located.

This study was conducted at the dispensary in Kileo, a village in Mwanga District, Kilimanjaro Region, Northern Tanzania. A dispensary is the basic unit in the Tanzanian health system that provides primary health care at the lowest facility level. The dispensary in Kileo is a government-owned health facility which opened in 1970s. The health facility serves two villages, Kileo and Kivulini, with populations of 2520 and 4255, respectively. Reproductive health and child health are among the services offered at the facility [8]. The area was chosen because of its history of high prevalence (36%) of urogenital schistosomiasis among women of reproductive age, which was reported more than 20 years ago [11]. Kileo and Kivulini are located in Kileo Ward (Figure 1). In these villages, the main economic activity is agriculture. The agricultural activities are performed in a traditional irrigation scheme, which receives water from Kivulini river [11].

### Study design and population

A hospital-based cross-sectional study involving quantitative methods was carried out between May and June 2021 to investigate the current burden of urogenital schistosomiasis among women of reproductive age, and to assess their knowledge and practices. The participants were recruited while visiting the dispensary for health services other than schistosomiasis. Participants aged 15–45 years at the time of data collection, who had lived in the area for at least 1 year were included in this study. Participants who reported that they had taken schistosomiasis treatment in the past 3 months, those who refused to provide written informed consent, those who refused to give a urine sample, and those who were seriously ill were excluded.

### Sample size estimation and sampling technique

The sample size for this study was calculated from a formula for cross-sectional surveys described by Creswell [3]. The study used prevalence of 36% for *S. haematobium* among women of reproductive age reported in a previous study conducted in the area [11], with a margin of error of 0.05, and standard deviation (SD) with 95% confidence intervals (CI) (1.96). Therefore, the estimated sample size for this study was 354 women of reproductive age. This study used convenience sampling, whereby study participants were recruited based on their availability. The participants were recruited serially as they presented at the dispensary.

### Sample collection and laboratory analysis

Prior to urine collection, study participants were oriented on the urine collection procedures. Wide-mouthed plastic containers with tight screw caps were pre-labelled with identification numbers, and distributed to study participants for the provision of approximately 50 mL of fresh urine. Urine samples were collected between 10:00 and 14:00 h, a period when peak egg shedding is expected. The urine samples were fixed immediately with 10% formalin to preserve the eggs of *S. haematobium*, and were taken to the parasitology laboratory at Muhimbili University of Health and Allied Sciences for examination. Urine samples were processed using the urine sedimentation technique, as described by the World Health Organization [19]. Briefly, conical centrifuge tubes were filled with 15 mL of urine, and centrifuged at 2000 *g* for 2 min. The supernatants were discarded by inverting the tubes, a few drops of sediment were placed on microscope slides, and coverslips were applied. Examination for the presence of *S. haematobium* eggs was undertaken using an Olympus CX 31 microscope (Tokyo, Japan).

### Questionnaire survey

A pre-tested structured questionnaire developed in English and then translated into Kiswahili was administered to all study participants by a trained interviewer. The questionnaire aimed to collect data on sociodemographic characteristics and schistosomiasis-related knowledge and practices of the study participants.

### Data analysis

Data collected were cleaned, coded and entered into Statistical Package for Social Sciences Version 20 (IBM Corp., Armonk, NY, USA) for analysis. Categorical data were summarized using frequency and proportion, and mean and SD were used for continuous data. Knowledge questions were computed as follows: 1 point was allocated for a correct response and 0 points were allocated for ‘don't know’ or incorrect responses. All knowledge questions had a total point score of 6 (if all answers were correct). A point score of <2 was considered poor, 2–4 average and ˃4 good. Practice questions were computed as follows: 1 point was allocated for a favourable practice and 0 points were allocated for an unfavourable (risky) practice. All practice questions had a total point score of 6 (if all answers were favourable practices). A point score of <2 was considered poor, 2–4 average and ˃4 favourable. Chi-squared test and Fisher's exact test were used to test associations between participants’ knowledge/practices and sociodemographic characteristics and schistosomiasis status. *P*<0.05 was considered to indicate significance.

## Results

### Sociodemographic characteristics of study participants

In total, 216 women of reproductive age (response rate 61%) agreed to participate in the study. The mean age of study participants was 19.5 (SD 6.1, range 15–45) years, with the majority (56.9%) in the <18 years age group. More than half of the participants were students (56.5%), and had lived in the village for >5 years (53.7%). The majority (95.8%) of study participants had received formal education (at least primary school level).

### Prevalence of urogenital schistosomiasis among study participants

The overall prevalence of urogenital schistosomiasis (S. haematobium) was 2.3% (95% CI 0.5–4.6%). Table 1 shows the proportion of participants infected with urogenital schistosomiasis based on sociodemographic characteristics. The prevalence was higher among participants aged ≥18 years (3.2%), those who had lived in the village for >5 years (2.6%), individuals without formal education (11.1%) and housewives (7.4%). However, the differences were not significant (*P*˃0.05).

**Table 1:** Sociodemographic characteristics and prevalence of urogenital schistosomiasis among the study participants

Sociodemographic characteristics			Urogenital schistosomiasis		
Characteristic	*n*	% (95% CI)	Infected	% (95% CI)	*P*-value
Age group					
≥18 years	93	43.1 (36.6–49.5)	3	3.2 (0.0–7.1)	0.370^a^
<18 years	123	56.9 (50.5–63.4)	2	1.6 (0.0–4.2)	
Time living in the village					
˃5 years	116	53.7 (47.2–60.2)	3	2.6 (0.0–5.9)	0.851^a^
1–5 years	88	40.7 (33.8–46.8)	2	2.3 (0.0–5.5)	
<1 year	12	5.6 (2.8–8.8)	0	0.0	
Level of education					
Formal education	207	95.8 (93.1–98.1)	4	1.9 (0.5–4.0)	0.193^a^
No formal education	9	4.2 (1.9–6.9)	1	11.1 (0.0–33.3)	
Occupation					
Housewife	27	12.5 (8.3–17.1)	2	7.4 (0.0–19.0)	0.089^a^
Peasant	41	19 (13.9–24.5)	1	2.4 (0.0–7.7)	
Student	122	56.5 (49.5–63.0)	2	1.6 (0.0–4.3)	
Petty business	26	12 (7.9–16.2)	0	0.0	

CI, confidence interval.

a Fisher's exact test.

### Awareness and knowledge of urogenital schistosomiasis among women of reproductive age

According to the findings, the majority of study participants (92.1%) were aware of urogenital schistosomiasis (Table 2). Of those who had heard of the disease, 55.8% said they had learned about it from health professionals. The majority of participants (86.1%) reported having some knowledge about urogenital schistosomiasis. However, only 15.6% understood that schistosomiasis is caused by parasitic worms. Only a small percentage (6.5%) knew that swimming in infested water increases the risk of transmission of schistosomiasis. A surprising majority (78%) knew that prolonged water contact is associated with transmission of schistosomiasis. Likewise, 62% of the participants knew that snails either transmit or support the life cycle of schistosoma parasites. The majority (97.3%) of participants were able to correctly name at least one symptom of urogenital schistosomiasis, while half (51.1%) knew that praziquantel is the drug used for treatment of urogenital schistosomiasis. Wearing gumboots in water was the preventive measure for schistosomiasis transmission mentioned by many (68.3%) participants. Overall, approximately one-quarter (26.9%) of all participants who reported having knowledge of urogenital schistosomiasis had good knowledge about the disease.

**Table 2:** Awareness and knowledge about urogenital schistosomiasis among women of reproductive age

Awareness/knowledge item	Total, *n* (%, 95% CI)	Urogenital schistosomiasis		
		Negative (%)	Positive (%)	*P*-value
Heard about schistosomiasis (*n*=216)				
Yes	199 (92.1, 88.4–95.4)	194 (97.5)	5 (2.5)	0.661^a^
Source of information (*n*=199)				
Television	30 (15.1, 10.4–20.3)	29 (96.7)	1 (3.3)	0.919^a^
Health expert	111 (55.8, 49.2–62.6)	108 (97.3)	3 (2.7)	
Radio	30 (15.1, 10.0–19.7)	30 (100.0)	0 (0.0)	
Other	28 (14.1, 9.7–18.9)	27 (96.4)	1 (3.6)	
Know anything about schistosomiasis (*n*=216)				
Yes	186 (86.1, 81.5–90.3)	182 (97.8)	4 (2.2)	0.530^a^
Causative agent of schistosomiasis (*n*=186)				
Virus, bacteria or fungus	114 (61.3, 54.1–68.3)	111 (97.4)	3 (2.6)	1.000^a^
Worms	29 (15.6, 10.6–21.0)	29 (100.0)	0 (0.0)	
I don't know	43 (23.1, 16.9–29.3)	42 (97.7)	1 (2.3)	
Risk of transmission of urogenital schistosomiasis (*n*=186)				
Walking bare foot	15 (8.1, 4.3–12.6)	13 (86.7)	2 (13.3)	0.018^a^
Swimming in polluted water	12 (6.5, 3.2–10.2)	11 (91.7)	1 (8.3)	
Prolonged water contact	145 (78.0, 72.2–83.6)	144 (99.3)	1 (0.7)	
I don't know	14 (7.5, 3.9–11.2)	14 (100.0)	0 (0.0)	
Relationship between snails and urogenital schistosomiasis (*n*=186)				
Transmit schistosomiasis	41 (22.0, 16.4–28.3)	41 (100.0)	0 (0.0)	0.234^a^
Support the life cycle of the parasite	75 (40.3, 32.8–47.5)	74 (98.7)	1 (1.3)	
No relationship	28 (15.1, 20.2–20.3)	26 (92.9)	2 (7.1)	
I don't know	42 (22.6, 16.9–28.6)	41 (97.6)	1 (2.4)	
Symptoms of urogenital schistosomiasis (*n*=186)				
Haematuria	98 (52.7, 45.2–59.9)	96 (98.0)	2 (2.0)	0.104^a^
Pain during micturation	83 (44.6, 37.2–52.4)	82 (98.8)	1 (1.2)	
Skin rashes	1 (0.5, 0.0–1.7)	1 (100.0)	0 (0.0)	
I don't know	4 (2.2, 0.5–4.4)	3 (75.0)	1 (25.0)	
Treatment of urogenital schistosomiasis (*n*=186)				
Traditional healers	12 (6.5, 3.2–10.2)	11 (92.7)	1 (8.3)	0.044^a^
Praziquantel	95 (51.1, 43.4–58.0)	95 (100.0)	0 (0.0)	
Self-recovering	37 (19.9, 14.2–25.8)	35 (94.6)	2 (5.4)	
I don't know	42 (22.6, 16.6–28.9)	41 (97.6)	1 (2.4)	
Prevention against urogenital schistosomiasis (*n*=186)				
Drinking safe water	20 (10.8, 6.3–15.2)	19 (95.0)	1 (5.0)	0.143^a^
Wearing gumboots when in water	127 (68.3, 61.9–74.6)	126 (99.2)	1 (0.8)	
Avoid hand shaking	1 (0.5, 0.0–1.7)	1 (100.0)	0 (0.0)	
I don't know	38 (20.4, 14.9–26.1)	36 (94.7)	2 (5.3)	
Overall knowledge (*n*=186)				
Poor	9 (4.8, 2.1–8.3)	7 (77.8)	2 (22.2)	0.020^a^
Average	127 (68.3, 61.0–75.1)	125 (98.4)	2 (1.6)	
Good	50 (26.9, 20.7–33.5)	50 (100.0)	0 (0.0)	

a Fisher's exact test.

Urogenital schistosomiasis was significantly higher in participants who reported water contact with bare feet as a risk factor for transmission of urogenital schistosomiasis, in participants who reported that the best treatment of urogenital schistosomiasis is through traditional healers, and in participants with a poor overall level of knowledge (*P*<0.05).

### Awareness and knowledge of urogenital schistosomiasis among women of reproductive age by sociodemographic characteristics

Table 3 shows descriptions of awareness and knowledge of urogenital schistosomiasis by sociodemographic characteristics. Length of residency in the village was significantly associated with awareness (*P*=0.021). When compared with participants aged ≥18 years, those aged <18 years were nearly 15% more likely to know that prolonged water contact is a risk factor for transmission of urogenital schistosomiasis (*P*<0.001). The proportion of women who knew that haematuria is a symptom of urogenital schistosomiasis was 64.2% in women aged <18 years and 46.2% in women aged ≥18 years (*p*<0.001). Furthermore, compared with those aged ≥18 years, more than half (52.2%) of women aged <18 years knew that pain during micturition is one of the symptoms of urogenital schistosomiasis (*P*<0.001). Women aged <18 years had significantly more knowledge about urogenital schistosomiasis treatment (62.2%) than those aged ≥18 years (31.3%, *P*<0.001).

**Table 3:** Urogenital schistosomiasis awareness and knowledge among women of reproductive age by sociodemographic characteristics

Awareness/knowledge item	Age group (%)			Time lived in the village (%)				Level of education (%)			Occupation (%)				
	<18 years	≥18 years	*P*-value	<1 year	1–5 years	˃5 years	*P*-value	Informal	Formal	*P*-value	Peasant	Student	Petty business	Housewife	*P*-value
Heard about schistosomiasis (*n*=216)															
Yes	121 (98.4)	78 (83.9)	<0.001	9 (75.0)	85 (96.6)	105 (90.5)	0.021	9 (100.0)	190 (91.2)	0.471^a^	38 (92.7)	122 (100.0)	19 (73.1)	20 (74.1)	0.001^a^
Source of information (*n*=199)															
Television	24 (19.8)	6 (7.7)	<0.001	1 (11.1)	19 (22.4)	10 (9.5)	0.021^a^	0 (0.0)	30 (15.8)	0.009^a^	3 (7.8)	24 (19.7)	2 (10.5)	1 (5.0)	0.001^a^
Health expert	73 (60.3)	38 (48.7)		7 (77.8)	50 (58.8)	54 (51.4)		2 (22.2)	109 (57.4)		19 (50.0)	75 (61.5)	10 (52.6)	7 (35.0)	
Radio	16 (13.2)	14 (17.9)		0 (0.0)	9 (10.6)	21 (20.0)		3 (33.3)	27 (14.2)		8 (21.1)	15 (12.3)	5 (26.4)	2 (10.0)	
Other	8 (6.6)	20 (25.6)		1 (11.1)	7 (8.2)	20 (19.0)		4 (44.5)	24 (12.6)		8 (21.1)	8 (6.5)	2 (10.5)	10 (50.0)	
Know anything about schistosomiasis (*n*=216)															
Yes	119 (96.7)	67 (72.0)	<0.001	8 (66.7)	80 (90.9)	98 (84.5)	0.057	8 (88.9)	178 (86.0)	0.637^a^	34 (82.9)	120 (98.4)	16 (61.5)	16 (59.3)	0.001^a^
Causative agent of schistosomiasis (*n*=186)															
Worms	19 (16.0)	10 (14.9)	<0.001	0 (0.0)	9 (11.2)	20 (20.4)	<0.001^a^	1 (12.5)	28 (15.7)	0.178^a^	7 (20.6)	17 (14.2)	3 (18.8)	2 (12.5)	<0.001^a^
Virus, bacteria or fungus	92 (77.3)	22 (32.8)		7 (87.5)	65 (81.2)	42 (42.9)		3 (37.5)	111 (62.4)		9 (26.5)	95 (79.2)	6 (37.5)	4 (25.0)	
I don't know	8 (6.7)	35 (52.3)		1 (12.5)	6 (7.5)	36 (36.7)		4 (50.0)	39 (21.9)		18 (52.9)	8 (6.7)	7 (43.7)	10 (62.5)	
Walking bare foot	12 (10.1)	3 (4.1)	<0.001	1 (12.5)	8 (10.0)	6 (6.1)	0.136^a^	1 (12.5)	14 (7.9)	0.001^a^	3 (8.8)	11 (9.2)	1 (6.2)	0 (0.0)	0.001^a^
Swimming in polluted water	7 (5.9)	5 (7.5)		1 (12.5)	4 (5.0)	7 (7.1)		2 (25.0)	10 (5.6)		0 (0.0)	8 (6.7)	1 (6.2)	3 (18.8)	
Prolonged water contact	99 (83.2)	46 (68.7)		6 (75.0)	66 (82.5)	73 (74.5)		1 (12.5)	144 (80.9)		25 (73.5)	101 (84.2)	11 (68.8)	8 (50.0)	
I don't know	1 (0.8)	13 (19.4)		0 (0.0)	2 (2.5)	12 (12.2)		4 (50.0)	10 (5.6)		6 (17.6)	0 (0.0)	3 (18.8)	5 (31.2)	
Relationship between snails and urogenital schistosomiasis (*n*=186)															
Transmit schistosomiasis	35 (29.4)	6 (9.0)	<0.001	1 (12.5)	23 (28.8)	17 (17.3)	<0.001^a^	1 (12.5)	40 (22.5)	0.004^a^	3 (8.8)	36 (30.0)	1 (6.2)	1 (6.2)	<0.001^a^
Support life cycle	62 (52.1)	13 (19.4)		5 (62.5)	46 (57.5)	24 (24.5)		0 (0.0)	75 (42.1)		7 (20.6)	63 (52.5)	5 (31.2)	0 (0.0)	
No relationship	16 (13.4)	12 (17.9)		1 (12.5)	7 (8.8)	20 (20.4)		4 (50.0)	24 (13.5)		5 (14.7)	16 (13.3)	2 (12.5)	5 (31.3)	
I don't know	6 (5.0)	36 (53.7)		1 (12.5)	4 (5.0)	37 (37.8)		3 (37.5)	39 (21.9)		19 (55.9)	5 (4.2)	8 (50.0)	10 (62.5)	
Symptoms of urogenital schistosomiasis (*n*=186)															
Haematuria	55 (46.2)	43 (64.2)	<0.001^a^	5 (62.5)	34 (42.5)	59 (60.2)	0.030^a^	5 (62.5)	93 (52.2)	0.786^a^	24 (70.6)	54 (45.0)	10 (62.5)	10 (62.5)	0.001^a^
Skin rashes	1 (0.8)	0 (0.0)		0 (0.0)	0 (0.0)	1 (1.0)		0 (0.0)	1 (0.6)		7 (20.6)	65 (54.2)	6 (37.5)	5 (31.3)	
Micturition	63 (52.9)	20 (29.9)		2 (25.0)	45 (56.2)	36 (36.7)		3 (37.5)	80 (44.9)		0 (0.0)	1 (0.8)	0 (0.0)	0 (0.0)	
I don't know	0 (0.0)	4 (6.0)		1 (12.5)	1 (1.2)	2 (2.0)		0 (0.0)	4 (2.2)		3 (8.8)	0 (0.0)	0 (0.0)	1 (6.2)	
Treatment of urogenital schistosomiasis (*n*=186)															
Traditional healers	8 (6.7)	4 (6.0)	<0.001	2 (25.0)	2 (2.5)	8 (8.2)	<0.001^a^	0 (0.0)	12 (6.7)	0.028^a^	1 (2.9)	8 (6.7)	1 (6.2)	2 (12.5)	
Praziquantel	74 (62.2)	21 (31.3)		4 (50.0)	51 (63.8)	40 (40.8)		1 (12.5)	94 (52.8)		14 (41.2)	76 (63.3)	5 (31.2)	0 (0.0)	
Self-recovering	31 (26.1)	6 (9.0)		1 (12.5)	24 (30.0)	12 (12.2)		2 (25.0)	35 (19.7)		3 (8.8)	31 (25.8)	1 (6.2)	2 (12.5)	
I don't know	6 (5.0)	36 (53.7)		1 (12.5)	3 (3.8)	38 (38.8)		5 (62.5)	37 (20.8)		16 (47.1)	5 (4.2)	9 (56.2)	12 (75.0)	
Prevention against urogenital schistosomiasis (*n*=186)															
Drink safe water	16 (13.4)	4 (6.0)	<0.001^a^	0 (0.0)	8 (10.0)	12 (12.2)	0.001^a^	1 (12.5)	19 (10.7)	0.752^a^	2 (5.9)	16 (13.3)	1 (6.2)	1 (6.2)	0.001^a^
Wear boots when in water	92 (77.3)	35 (52.2)		6 (75.0)	66 (82.5)	55 (56.1)		5 (62.5)	122 (68.5)		19 (55.9)	94 (78.3)	8 (50.0)	6 (37.5)	
Avoid hand shaking	1 (0.8)	0 (0.0)		0 (0.0)	0 (0.0)	1 (1.0)		0 (0.0)	1 (0.6)		0 (0.0)	1 (0.8)	0 (0.0)	0 (0.0)	
I don't know	10 (8.4)	28 (41.8)		2 (25.0)	6 (7.5)	30 (30.6)		2 (25.0)	36 (20.2)		13 (38.2)	9 (7.6)	7 (43.8)	9 (56.2)	
Overall knowledge (*n*=186)															
Poor	2 (1.7)	7 (10.4)	0.011	1 (12.5)	4 (5.0)	4 (4.1)	0.072^a^	2 (25.0)	7 (3.9)	0.023^a^	2 (5.9)	2 (1,7)	1 (6.2)	4 (25.0)	0.002^a^
Average	80 (67.2)	47 (70.1)		6 (75.0)	47 (58.8)	74 (75.5)		6 (75.0)	121 (68.0)		24 (70.6)	79 (65.8)	12 (75.0)	12 (75.0)	
Good	37 (31.1)	13 (19.4)		1 (12.5)	29 (36.2)	20 (20.4)		0 (0.0)	50 (28.1)		8 (23.8)	39 (32.5)	3 (18.8)	0 (0.0)	

a Fisher's exact test.

When compared with other occupational groups, the proportion of students who had heard of schistosomiasis was higher (100%, *P*<0.001). Awareness of the risk of urogenital schistosomiasis transmission was higher among those with formal education than those without formal education (12.1%, *P*<0.001). Furthermore, when compared with other occupational groups, students had significantly higher knowledge about the risk of schistosomiasis transmission (84.2%, *P*<0.001).

Approximately 42% of women with formal education knew that snails support the life cycle of the schistosoma parasites that cause urogenital schistosomiasis. The proportion was higher among students (52.5%) compared with other occupational groups. Moreover, 50% of women without formal education responded that there is no relationship between snails and urogenital schistosomiasis (*P*<0.001). More than 50% of women with formal education knew that urogenital schistosomiasis can be treated using praziquantel, compared with 12.5% of women without formal education (*P*<0.001).

### Urogenital schistosomiasis practices among women of reproductive age

According to the findings, approximately one-third of participants (31.5%) used river water for various activities and practiced swimming (32.4%). Twenty-eight percent of participants said that they used river water for agricultural purposes. Only two (0.9%) of the participants admitted to urinating in water sources, and none of the participants reported not using the toilets at their workplace. In general, only 1.9% of participants had poor practices (Table 4).

**Table 4:** Urogenital schistosomiasis practices among women of reproductive age (*n*=216).

Practice item	Total, *n* (%, 95% CI)	Schistosomiasis		
		Negative (%)	Positive (%)	*P*-value
Source of water for domestic purposes				
River	68 (31.5, 25.5–37.5)	65 (95.6)	3 (4.4)	0.328^a^
Tap	98 (45.4, 38.9–51.9)	96 (98.0)	2 (2.0)	
Well	49 (22.7, 16.7–28.2)	49 (100.0)	0 (0.0)	
Rain	1 (0.5, 0.0–1.4)	1 (100.0)	0 (0.0)	
Water-related activity				
Domestic activities	15 (6.9, 3.7–11.1)	13 (86.7)	2 (13.3)	0.001^a^
Agriculture	61 (28.2, 21.8–34.3)	58 (95.1)	3 (4.9)	
None	140 (64.8, 58.3–71.8)	140 (100.0)	0 (0.0)	
Water contact with bare feet				
Yes	118 (54.6, 48.6–61.1)	115 (97.5)	3 (2.5)	0.587^a^
No	98 (45.4, 38.9–51.4)	96 (98.0)	2 (2.0)	
Swim in river water				
Yes	70 (32.4, 25.9–38.9)	65 (92.9)	5 (7.1)	0.003^a^
No	146 (67.6, 61.1–74.1)	146 (100.0)	0 (0.0)	
Urinate in water source				
Yes	2 (0.9, 0.0–2.3)	2 (100.0)	0 (0.0)	0.954^a^
No	214 (99.1, 97.7–100.0)	209 (99.1)	5 (2.3)	
Use toilets at workplace				
Yes	216 (100.0, 100.0–100.0)	211 (100.0)	5 (100.0)	NA
No	0	0 (0.0)	0 (0.0)	
Overall practices				
Poor	4 (1.9, 0.5–3.7)	2 (50.0)	2 (50.0)	0.001^a^
Average	94 (43.5, 37.0–50.0)	92 (97.9)	2 (2.1)	
Favourable	118 (54.6, 48.1–61.1)	117 (98.2)	1 (0.8)	

CI, confidence interval; NA, not applicable.

a Fisher's exact test.

Water contact during domestic activities was associated with a significantly higher risk of urogenital schistosomiasis (13.3%) compared with water contact during agricultural activities (4.9%, *P*=0.001). Similarly, the prevalence of urogenital schistosomiasis was significantly higher (7.1%) among women who swam in the river, whereas none of the women who did not swim in the river were infected (0%, *P*=0.003). In general, the prevalence of urogenital schistosomiasis was significantly higher among women with poor practices (50%) compared with women with average and favourable practices (2.1% and 0.8%, respectively; *P*=0.001).

### Urogenital schistosomiasis practices among women of reproductive age by sociodemographic characteristics

Table 5 provides a summary of urogenital schistosomiasis practices among women of reproductive age by sociodemographic characteristics. Women aged ≥18 years were more likely to use river water than women aged <18 years (45.2% vs 21.1%, respectively). However, the proportion of women conducting domestic activities in river water was two-fold higher among those aged <18 years compared with those aged ≥18 years (*P*<0.001). Similarly, women who had lived in the village for >5 years were more likely to use river water than those who had only lived there for a few years (*P*<0.001). Furthermore, 37.4% of women aged <18 years reported swimming in river water, compared with 25.8% of those aged ≥18 years.

**Table 5:** Urogenital schistosomiasis practices among women of reproductive age by sociodemographic characteristics

Practice item	Age group (%)			Time lived in the village (%)				Level of education (%)			Occupation (%)				
	<18 years	≥18 years	*P*-value	<1 year	1–5 years	˃5 years	*P*-value	Informal	Formal	*P*-value	Peasant	Student	Petty business	Housewife	*P*-value
Source of water for domestic purposes															
River	26 (21.1)	42 (45.2)	<0.001^a^	4 (33.3)	14 (15.9)	50 (43.1)	<0.001^a^	2 (22.2)	66 (31.9)	0.764^a^	19 (46.3)	25 (20.5)	12 (46.2)	12 (44.4)	<0.001^a^
Tap	74 (60.2)	24 (25.8)		8 (66.7)	59 (67.0)	31 (26.7)		4 (44.4)	94 (45.4)		8 (19.5)	75 (61.5)	7 (26.9)	8 (29.6)	
Well	22 (17.9)	27 (29.0)		0 (0.0)	14 (15.9)	35 (30.2)		3 (33.3)	46 (22.2)		14 (34.1)	21 (17.2)	7 (26.9)	7 (25.9)	
Rain	1 (0.8)	0 (0.0)		0 (0.0)	1 (1.1)	0 (0.0)		0 (0.0)	1 (0.5)		0 (0.0)	1 (0.8)	0 (0.0)	0 (0.0)	
Water-related activity in the river															
Domestic activities	11 (8.9)	4 (4.3)	<0.001	0 (0.0)	16 (18.2)	44 (37.9)	0.006	4 (44.4)	57 (27.5)	0.281	0 (0.0)	11 (9.0)	2 (7.7)	2 (7.4)	
Agriculture	17 (13.8)	44 (47.3)		1 (8.3)	16 (18.2)	44 (37.9)		1 (11.1)	14 (6.8)		32 (78.0)	15 (12.3)	5 (19.2)	9 (33.3)	
None	95 (77.2)	45 (48.4)		11 (91.7)	66 (75.0)	63 (54.3)		4 (44.4)	136 (65.7)		9 (22.0)	96 (78.7)	19 (73.1)	16 (59.3)	
Water contact with bare feet															
Yes	63 (51.2)	55 (59.1)	0.271	5 (41.7)	47 (53.4)	66 (56.9)	0.575	8 (88.9)	110 (53.1)	0.034^a^	30 (73.2)	61 (50.0)	12 (46.2)	15 (55.6)	0.053
No	60 (48.8)	38 (40.9)		7 (58.3)	41 (46.6)	50 (43.1)		1 (11.1)	97 (46.9)		11 (26.8)	61 (50.0)	14 (53.8)	12 (44.4)	
Swim in river water															
Yes	46 (37.4)	24 (25.8)	0.048	4 (33.3)	38 (43.2)	28 (24.1)	0.016	4 (44.4)	142 (68.6)	0.126	9 (22.0)	46 (37.7)	8 (30.8)	7 (25.9)	0.260
No	77 (62.6)	69 (74.2)		8 (66.7)	50 (56.8)	88 (75.9)		5 (55.6)	65 (31.4)		32 (78.0)	76 (62.3)	18 (69.2)	20 (74.1)	
Urinate in water source															
Yes	1 (0.8)	1 (1.1)	0.677^a^	0 (0.0)	1 (1.1)	1 (0.9)	0.923^a^	0 (0.0)	2 (1.0)	0.918^a^	1 (2.4)	1 (0.8)	0 (0.0)	0 (0.0)	
No	122 (99.2)	92 (98.9)		12 (100.1)	87 (98.9)	115 (99.1)		9 (100.0)	205 (99.0)		40 (97.6)	121 (99.2)	26 (100.0)	27 (100.0)	0.682
Use toilets at workplace or residence															
Yes	123 (100.0)	93 (100.0)	NA	12 (100.0)	88 (100.0)	116 (100.0)	NA	9 (100.0)	207 (100.0)	NA	41 (100.0)	122 (100.0)	26 (100.0)	27 (100.0)	NA
No	0 (0.0)	0 (0.0)		0 (0.0)	0 (0.0)	0 (0.0)		0 (0.0)	0 (0.0)		0 (0.0)	0 (0.0)	0 (0.0)	0 (0.0)	
Overall practices															
Poor	2 (1.6)	2 (2.2)	0.088^a^	0 (0.0)	2 (2.3)	2 (1.7)	0.473^a^	0 (0.0)	4 (1.9)	0.915^a^	2 (4.9)	2 (1.6)	0 (0.0)	0 (0.0)	0.231^a^
Average	46 (37.4)	48 (51.6)		6 (50.0)	32 (36.4)	56 (48.3)		4 (44.4)	90 (43.5)		22 (53.7)	46 (37.7)	14 (53.8)	12 (44.4)	
Favourable	75 (61.0)	43 (46.2)		6 (50.0)	54 (61.4)	58 (50.0)		5 (55.6)	113 (54.6)		17 (41.5)	74 (60.7)	12 (46.2)	15 (55.6)	

NA, not applicable.

a Fisher's exact test.

The proportion of women reporting water contact with bare feet was higher (88.9%) among women without formal education compared with those with formal education (53.1%, *P*=0.034). When compared with other water sources, many peasants (46.3%), petty businesses (46.2%) and housewives (44.4%) reported a preference for river water over other sources (*P*<0.001). The majority of peasants reported that they used river water during their agricultural activities (78.0%) and contacted the water with bare feet (73.2%).

## Discussion

Tanzania has the second highest (after Nigeria) prevalence of schistosomiasis in Africa [10]. Despite the high prevalence and persistence of urogenital schistosomiasis in Tanzania, there is little information on the current burden of urogenital schistosomiasis among women of reproductive age. The current study found that urogenital schistosomiasis transmission was low among women of reproductive age in Mwanga District. This indicates a significant decrease in urogenital schistosomiasis compared with a previous study conducted in the same area approximately 20 years ago, in which 36% of women of reproductive age were diagnosed with urogenital schistosomiasis [11]. The observed decline could be attributed to previous interventions such as mass treatment, health education, sanitation improvement, and provision of access to safe water [12]. Similar low prevalence of urogenital schistosomiasis has been reported among women of reproductive age living in North-western Tanzania. However, the study in North-western Tanzania reported differences in the prevalence of urogenital schistosomiasis between villages within the same region [5]. Furthermore, a study in Kenya found that pregnant women are at increased risk of urogenital schistosomiasis, with prevalence ranging from 20.0% to 58.3%, while non-pregnant women had prevalence ranging from 12.5% to 42.9% [7].

This study found that the prevalence of urogenital schistosomiasis was two-fold higher in women who used river water compared with those who used tap water. The prevalence of urogenital schistosomiasis was also significantly higher among women who swam in river water, while none of the women who did not swim in river water had urogenital schistosomiasis. This indicates that people are at risk of infection when they use unsafe water [13,17]. Thus, in addition to mass chemotherapy, clean water supply and behavioral change interventions, such as avoiding domestic and recreational activities including swimming or fishing in infested water, can help to interrupt the transmission of schistosomiasis. Also, the current study found that water contact during domestic activities was associated with a higher risk of urogenital schistosomiasis compared with water contact during agricultural activities. Obviously, water contact during domestic activities is more frequent than water contact during agricultural activities in areas where irrigation is practiced periodically.

The majority of participants were knowledgeable about urogenital schistosomiasis and knew at least one symptom, but only 15% understood that schistosomiasis is caused by parasitic worms. Similar studies in Cameron and Nigeria found that adult participants were unaware of the causes of schistosomiasis [4,6]. The present study found that more than half of the participants were aware that snails play a role in the transmission of urogenital schistosomiasis, and that praziquantel is used to treat the disease. In contrast, Folefac *et al.* [6] and Dawaki *et al.* [4] reported that none of the participants recognized the role of snail vectors. Most of the participants knew that water contact is associated with exposure to urogenital schistosomiasis. However, very few seemed to associate swimming practices with urogenital schistosomiasis. Similarly, Dawaki *et al.* [4] found that only 27.9% of the respondents associated urogenital schistosomiasis with contact with contaminated water. This indicates a significant gap in knowledge about the transmission of schistosomiasis, which could easily be covered during mass drug distribution campaigns. In general, knowledge about urogenital schistosomiasis was higher among women aged <18 years compared with those aged ≥18 years. Many of the women aged <18 years could be students, possibly being taught about health education in schools.

In addition, knowledge about the risk of urogenital schistosomiasis transmission was significantly higher among women with formal education compared with those without formal education. Moreover, more than half of the women with formal education knew that urogenital schistosomiasis can be treated using praziquantel, compared with only 12% of the women without formal education. A similar study among the rural population in Yemen found that parents with formal education were more likely to have heard about schistosomiasis, know at least one symptom of schistosomiasis, and understand the role of snails as schistosomiasis vectors than uneducated women [15]. Furthermore, students were more likely to have heard about schistosomiasis and had significantly higher knowledge of urogenital schistosomiasis compared with other groups. However, both women with and without formal education had a very low understanding of the causes of schistosomiasis.

With regards to practices, women aged <18 years had favourable practices compared with women aged ≥18 years. Swimming and domestic activities in rivers, on the other hand, were more popular among women aged <18 years than among those aged ≥18 years. Similar findings were recorded among rural communities in Kano State [4]. Likewise, women who had lived in the village for >5 years were more likely to use river water compared with those who had only lived in the village for a few years. In addition, water contact with bare feet was higher among women without formal education compared with women with formal education. However, the source of water used for domestic purposes, water-related activities, and swimming in river water were not significantly related to the level of education. These findings are in contrast to a study in Zimbabwe [9], where educated women were 40% less likely to use unsafe water sources for domestic purposes compared with uneducated women. However, this could depend on the availability of safe water in the area.

## Conclusions and recommendations

This study demonstrated persistence and low transmission of urogenital schistosomiasis among women of reproductive age in Kileo, Mwanga District. Despite high general awareness about urogenital schistosomiasis among women, there are still knowledge gaps regarding its causes and risk factors. As targets progress from morbidity control to schistosomiasis elimination as a public health problem, the inclusion of appropriate health education and behavioural change interventions targeting women, particularly at reproductive health and child health clinics, and the community during preventive chemotherapy campaigns is essential to interrupt the transmission of schistosomiasis.

### Study limitations

The prevalence of urogenital schistosomiasis may have been underestimated in this study. This is because participants at the study setting (dispensary) were more likely to seek medical attention, and thus may have been treated for schistosomiasis during previous visits. Furthermore, the diagnostic test used was less sensitive. However, all sediments were examined in order to increase sensitivity.

## Conflict of interest statement

None declared.
